# Distinct Aging Effects on Motion Repulsion and Surround Suppression in Humans

**DOI:** 10.3389/fnagi.2017.00363

**Published:** 2017-11-07

**Authors:** Hu Deng, Weiying Chen, Shenbing Kuang, Tao Zhang

**Affiliations:** ^1^State Key Laboratory of Brain and Cognitive Science, Institute of Psychology, Chinese Academy of Sciences, Beijing, China; ^2^Department of Psychology, University of Chinese Academy of Sciences, Beijing, China

**Keywords:** aging, motion repulsion, surround suppression, local mutual inhibition, global inter-areal inhibition

## Abstract

Elderly exhibit accumulating deficits in visual motion perception, which is critical for humans to interact with their environment. Previous studies have suggested that aging generally reduces neuronal inhibition in the visual system. Here, we investigated how aging affects the local intra-cortical inhibition using a motion direction discrimination task based on the motion repulsion phenomenon. Motion repulsion refers to the phenomenon by which observers overestimate the perceived angle when two superimposed dot patterns are moving at an acute angle. The misperception has been interpreted as local mutual inhibition between nearby direction-tuned neurons within the same cortical area. We found that elderly exhibited much stronger motion repulsion than young adults. We then compared this effect to how aging affects the global inter-cortical inhibition by adopting the surround suppression paradigm previously used by Betts et al. (2005). We found that elderly showed less change in the discrimination threshold when the size of a high-contrast drifting Gabor was increased, indicating reduced surround suppression compared to young adults. Our results indicate that aging may not always lead to a decrease of neuronal inhibition in the visual system. These distinct effects of aging on inhibitory functions might be one of the reasons that elderly people often exhibit deficits of motion perception in a real-world situation.

## Introduction

As the human lifespan has been continuously increasing over the past century, aging has become a worldwide phenomenon. The quality of life can be seriously affected by the aging process, because various perceptual abilities generally decline with aging. Because visual motion perception is essential for humans to interact with the environment, aging-induced accumulating deficits in visual motion perception can have profound impacts on normal life, such as inducing a higher risk of car accidents (Boot et al., 2014). Previous studies have shown that elderly exhibit accumulating deficits in the form of reduced sensitivity of motion detection and discrimination (Bennett et al., 2007), impaired perceptual efficiency (Bower and Andersen, 2012), and elevated thresholds for motion speed perception (Snowden and Kavanagh, 2006). Interestingly, visual capabilities do not generally decline with normal aging. For example, it has been reported that directional sensitivity of the retina and color vision-related perceptual constancies exhibit little or no alteration with aging (Enoch et al., 1999). Aging also affects various types of motion processing in different ways. For example, aging increases the thresholds for translational motion but not for radial flow perception (Billino et al., 2008). As the worldwide aged population rapidly expands, it is urgent to determine the underlying mechanisms of age-related visual impairments and to develop interventions to improve the quality of life of elderly (Owsley, 2011).

Previous studies have shown that age-related visual declines may be caused by degenerative neuronal functions in the visual cortex (Andersen, 2012). Such evidence has been reported based on neural representations of visual stimulus selectivity (Liang et al., 2010; Fu et al., 2013), speed (Yang et al., 2009), contrast sensitivity (Wang et al., 2014) and heading direction (Kavcic et al., 2013). It is well known that the inhibitory system plays a crucial role in cortical function (Isaacson and Scanziani, 2011). Therefore, the declined visual functions of elderly may be consequences of abnormal cortical inhibition. One strong piece of evidence is that administration of GABA and the GABAa receptor agonist muscimol to aged monkeys led to improved neural representations in the V1 area (Leventhal et al., 2003).

Two major inhibitory mechanisms mediate cortical functions. One is the local mutual inhibition, which is the inhibitory neuronal connections within the same cortical region. The other is the global inter-areal inhibition. “Global inter-areal inhibition” refers to the interaction between excitatory feedback connections and inhibitory horizontal connections (Angelucci and Bullier, 2003). Two perceptual phenomena are associated with these inhibitory mechanisms. Motion repulsion refers to the phenomenon by which observers overestimate the perceived angle when two superimposed dot patterns are moving at an acute angle (Marshak and Sekuler, 1979; Rauber and Treue, 1999). The misperception has been interpreted as being caused by local mutual inhibition between nearby direction-tuned neurons within the same cortical region (Marshak and Sekuler, 1979; Hiris and Blake, 1996; Rauber and Treue, 1999; Matthews et al., 2000; Perry et al., 2014). In motion direction discrimination tasks, participants must discriminate the direction of drifting Gabors of different contrasts and sizes. For high-contrast drifting Gabors, the motion direction discrimination threshold increases with increasing Gabor size (Tadin et al., 2003), which is considered as the behavioral manifestation of center-surround suppression (Betts et al., 2005, 2009). Neural surround suppression is considered to be primarily mediated by global inter-areal inhibition from higher-level cortical regions to the primary visual cortex (Angelucci and Bullier, 2003; Schwabe et al., 2006; Nassi et al., 2013).

It has been reported that elderly exhibit declined global inter-areal inhibition (Betts et al., 2005, 2009). However, how aging affects local mutual inhibition remains unclear. To clarify whether aging has a unified impact on cortical inhibition, we adopted motion repulsion and motion direction discrimination tasks in this study to compare aging effects on different inhibitory mechanisms.

## Materials and Methods

All subjects had normal or corrected-to-normal vision, and the old subjects underwent an eye examination before participating in the study to exclude eye diseases such as cataracts, amblyopia and macular degeneration. Old subjects were also excluded for cognition impairments based on the Mini Mental State Examination (normal range: 27–30, mean score: 28.8 points; Folstein et al., 1975). All experimental protocols were approved by the Ethics Review Committee of Institute of Psychology, Chinese Academy of Sciences. All experimental procedures described below were conducted in accordance with institutional ethical guidelines, and written informed consent was obtained from all subjects.

The visual stimuli and experimental control stimuli were generated in Matlab using the Psychophysics Toolbox extension (Brainard, 1997; Pelli, 1997; Kleiner et al., 2007).

## Experiment 1

### Participants

Eleven naive young participants (5 females and 6 males, mean age ± SD = 24.0 ± 1.31) and 11 naive old participants (6 females and 5 males, mean age ± SD = 75.38 ± 3.62) completed the experiment.

### Procedure

Experiments were performed in a dark, quiet room. Participants were seated 47 cm from an LCD computer monitor (19 inch; screen resolution: 1440 × 900; refresh rate: 60 Hz), and their heads remained in a fixed position via the aid of a chinrest. The monitor was equipped with an Infrared Multi-Point Touch Screen, which could record the touch positions that the participants reported by touching the screen.

**Figure 1:**
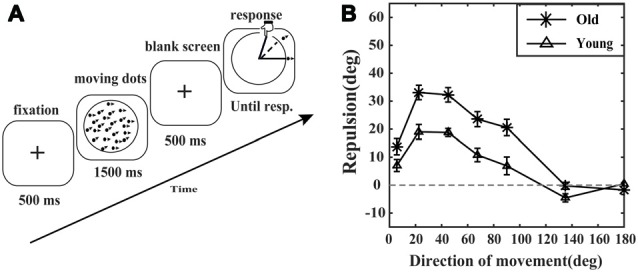
Motion repulsion experiment paradigm and results. **(A)** A schematic of the motion repulsion experiment. Each trial began with a fixation point followed by two superimposed moving random dot kinematograms (RDKs). The reference RDKs always moved horizontally to the right. The moving direction of the target RDKs was varied trial-by-trial. The observer was required to report the perceived moving direction of the target RDKs by touching the corresponding point on the circle with his or her finger. **(B)** Motion repulsion plotted as a function of the moving direction of the target RDKs. Motion repulsion was calculated as the difference between the perceived and actual angles of the target RDKs. Data points represent the average motion repulsion of the elderly (asterisk marker, *N* = 11) and young adults (triangle marker, *N* = 11). Error bars represent the SEM.

The procedure used to generate the stimuli was similar to a previous study (Kuang and Zhang, 2014). The reference random dot kinematograms (RDKs), dot diameter = 0.1 degrees, patch radius = 4.9 degrees, dot number = 200, coherence = 100%, dot speed = 3 degrees/s, always moved horizontally to the right. The target RDKs moved at varying directions among trials. The actual angles between two motion directions were 5.6, 22.5, 45, 67.5, 90, 135 and 180 degrees. Each participant was required to complete 16 blocks of trials. Trials in each block were randomly interleaved. Each trial began with a black fixation cross (diameter = 0.75 degrees) centered on a gray screen. After 500 ms, a circular patch containing two superimposed RDKs appeared on the center of the screen for 500 ms, followed by a black screen for 500 ms. Then, a circle appeared on the center of the screen, and the participant was required to report the perceived moving direction of the target RDKs by touching the corresponding position on the circle, which constructed a line from the center of the circle to the point the participant touched (Figure 1A).

### Analysis

The amount of motion repulsion was defined as the perceived moving direction minus the actual moving direction of target RDKs. The motion repulsion differences between young adults and elderly were analyzed using a two-way mixed-design ANOVA with direction (5.6°, 22.5°, 45°, 67.5°, 90°, 135° and 180°, within-subject factor) and age (Old vs. Young, between-subject factor) in SPSS 19.0.

## Experiment 2

### Participants

Eight healthy young participants (2 females and 6 males, mean age ± SD = 21.88 ± 1.89) and eight healthy old participants (6 females and 2 males, mean age ± SD = 72.5 ± 4.21) completed Experiment 2.

### Stimuli and Procedure

**Figure 2:**
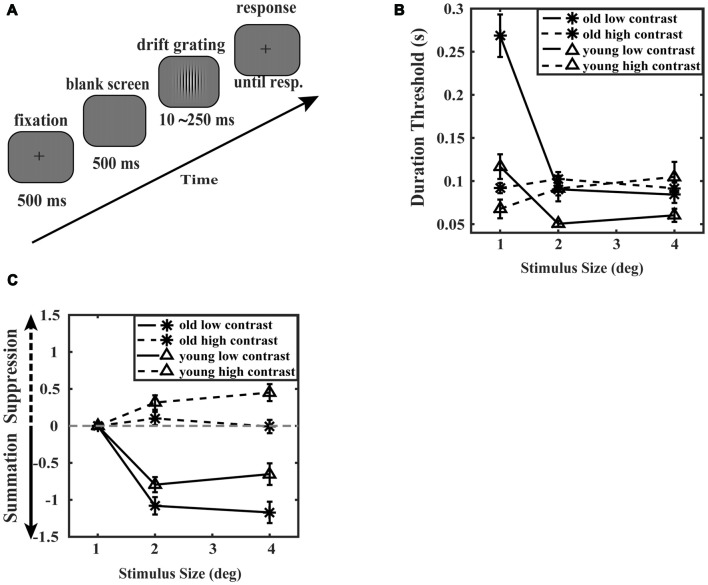
Surround suppression experiment paradigm and results. **(A)** A schematic of the surround suppression experiment. Each trial began with a fixation point followed by a blank screen for 500 ms. A drifting Gabor stimulus (1 cyc deg^−1^ sine wave Gabor drifting at a rate of 2 deg s^−1^) was then presented for various durations. After the stimulus disappeared, a fixation point was presented on the screen. The observer was required to report the direction of the drift of the Gabor as left or right by pressing one of the two response keys. The stimulus duration was operated by a 3-up 1-down staircase procedure. Reporting time was not limited. **(B)** Discrimination thresholds plotted as a function of the drifting Gabor size. Thresholds were calculated as the stimulus duration required 79.4% accuracy of trials. The dashed lines represent the high contrast condition and the solid lines indicate the low contrast condition. Data points represent the average motion repulsion of the elderly (asterisk marker, *N* = 8) and the young adults (triangle marker, *N* = 8). Error bars represent the SEM. **(C)** Mean surround suppression indexes plotted as a function of stimulus size. Surround suppression index (SI) was defined as the log difference between thresholds of each of the two larger stimuli (2 and 4 degrees) and thresholds of the 1 degree stimulus at low or high contrast. Asterisk markers represent the mean surround suppression indexes of the elderly, while triangle markers correspond to the young adults. The solid lines represent the mean surround suppression indexes at low contrast, and the dashed lines represent the mean surround suppression indexes at high contrast. Error bars represent the SEM.

Experiments were performed in a dark, quiet room. Stimuli were displayed on a 20-inch Sony CRT computer monitor with 800 × 600 resolution and a refresh rate of 100 Hz. The maximum luminance was 29 cd/m^2^. Participants were seated 57 cm from the display, and their heads remained in a fixed position via the aid of a chinrest. All behavioral responses were recorded using a Logitech Classic Keyboard. The visual stimuli and experimental procedure (Figure 2A) were similar to previous studies (Tadin et al., 2003; Betts et al., 2005; Golomb et al., 2009). Stimulus contrast was modulated by a two-dimensional Gaussian envelope, either 5% or 90%. The stimulus size was 1, 2, or 4 degrees of the visual angle. The experiment was designed in blocks by a combination of stimulus contrast and size, and each participant completed six blocks. The order of the six conditions was randomly balanced. There were two interleaved staircase procedures in every block, and each converged to the stimulus duration needed to produce 79.4% correct performance. Both staircases started at 250 ms, with an initial step size of 40 ms, switched to a step size of 20 ms after two reversals, and switched to a final step size of 10 ms after the fourth reversal. The block ended after 10 reversals, and the total trials were limited to 150.

### Analysis

The duration threshold was calculated as the mean of the final 6 reversal points, and the thresholds from the two staircases are averaged. Suppression indexes were calculated for each individual, and the differences of suppression indexes between the young group and the old group at all contrast levels were evaluated by a two-way mixed-design ANOVA analysis with stimulus size (1°, 2° and 4°, within-subject factor) and age (Old vs. Young, between-subject factor) in SPSS 19.0. The same ANOVA analysis were conducted for the raw thresholds.

## Results

### Experiment 1: Motion Repulsion

To investigate aging effects on motion repulsion, we presented young and old observers with two RDKs moving in different directions (for details, see “Materials and Methods” section). In previous studies (Marshak and Sekuler, 1979; Rauber and Treue, 1999), the visual angle between the perceived motion direction of target RDKs and reference RDKs was generally larger than the actual angle in both the young and old groups (Figure 1B), which is the behavioral correlation of the repulsive interactions between motion directions. This is the so-called motion repulsion effect. As shown in Figure 1B, the motion repulsion effects for both the young group and the old group first increased and then gradually decreased as the included angle varied from 5.6 to 180 degrees. In detail, for the young group, significant motion repulsion effects (greater than 0) were observed at 5.6, 22.5, 45, 65 and 135 (less than 0) degrees (*t*_(10)_ = 3.268, 7.239, 12.897, 4.465, −3.109; all corrected *p* < 0.05, Bonferroni-Holm corrected *t*-test), but not at 90 degree or 180 degree (*t*_(10)_ = 2.201, 1.091; all corrected *p* > 0.05, Bonferroni-Holm corrected *t*-test). For the old group, significant motion repulsion effects (greater than 0) were observed at 5.6, 22.5, 45, 65 and 90 degrees (*t*_(10)_ = 4.765, 12.762, 12.498, 8.741, 7.001; all corrected *p* < 0.05, Bonferroni-Holm corrected *t*-test), not at the 135 degree or 180 degree (*t*_(10)_ = −0.183, −1.070; all corrected *p* > 0.05, Bonferroni-Holm corrected *t*-test).

Comparing the motion repulsion effects between the young group and the old group by a two-way ANOVA, we found significant main effects of age (*F*_(1,20)_ = 30.583, *p* = 0.000, $\eta_{\text{p}}^{2}$ = 0.605) and direction (*F*_(6,120)_ = 56.020, *p* = 0.000, $\eta_{\text{p}}^{2}$ = 0.737), and there was a significant interaction of age and direction (*F*_(6,120)_ = 4.230, *p* = 0.003, $\eta_{\text{p}}^{2}$ = 0.175). Simple effect analysis showed that the motion repulsion effects at the 22.5, 45, 65, 90 and 135 degrees (all *p* < 0.05) were significantly different between the young group and the old group. As the age-related enhancement of overestimating the included angle varied across different directions, we further conducted a series of *post hoc* analysis (Unpaired *t*-tests, Bonferroni-Holm correction) to compare the motion repulsion effects between the young group and the old group at each direction. The results showed that the motion repulsion effects for the elderly at 22.5, 45, 65 and 90 degrees (*t*_(20)_ = 3.810, 4.540, 3.511, 3.150, *p* = 0.006, 0.001, 0.010, 0.020) were stronger than those of young adults, but not at 5.6 degree, 135 degree, or 180 degree (*t*_(20)_ = 1.860, 2.251, −1.321, *p* = 0.154, 0.107, 0.200).

## Experiment 2

### Suppression Index of Direction Discrimination

In comparing the various effects of aging on global inter-areal inhibition, we conducted an experiment previously described by Betts et al. (2005). Young participants and old participants were required to discriminate the motion direction of a drifting Gabor at two contrast levels and three stimulus sizes (for details, see “Materials and Methods” section). Discrimination thresholds were defined by the stimulus duration required to correctly perform the task for 79.4% of trials. As shown in Figure 2B, at high contrast, an ANOVA analysis of the discrimination thresholds revealed that there was a significant main effect of stimulus size (*F*_(2,28)_ = 5.696, *p* = 0.008, $\eta_{\text{p}}^{2}$ = 0.289), and the main effect of age was not significant (*F*_(1,14)_ = 0.260, *p* = 0.622, $\eta_{\text{p}}^{2}$ = 0.018). A significant interaction between stimulus size and age was observed (*F*_(2,28)_ = 4.801, *p* = 0.016, $\eta_{\text{p}}^{2}$ = 0.255). Simple effect analysis showed that the discrimination thresholds of small stimulus (1 degree) were not significantly different between the elderly and the young subjects (*F*_(1,14)_ = 3.840, *p* = 0.070, $\eta_{\text{p}}^{2}$ = 0.215). Also, another simple effect analysis showed that the thresholds of all stimuli size were not significantly different in the old group (*F*_(2,13)_ = 1.059, *p* = 0.375, $\eta_{\text{p}}^{2}$ = 0.140). *Post hoc* analysis (Bonferroni-Holm corrected *t*-test) revealed that the thresholds of the old subjects were not significantly different from the young subjects in all stimulus size conditions (1, 2, 4 degrees: *t*_(14)_ = 1.960, 0655, −0707, *p* = 0.211, 0.982, 0.982). These results suggest that the elderly and the young subjects have similar motion sensitivity of small moving stimulus. As shown in Figure 2B, the thresholds of the young adults increased with increasing stimulus size (*F*_(2,13)_ = 8.534, *p* = 0.004, $\eta_{\text{p}}^{2}$ = 0.568), which is a phenomenon known as surround suppression effect. But the thresholds of the old group did not increase with increasing stimulus size (*F*_(2,13)_ = 1.059, *p* = 0.375, $\eta_{\text{p}}^{2}$ = 0.140), indicating a decrease in surround suppression effect. At low contrast, discrimination thresholds of two groups declined with increasing stimulus size (*F*_(2,28)_ = 66.850, *p* = 0.000, $\eta_{\text{p}}^{2}$ = 0.827), which indicates a spatial summation effect.

We calculated the surround suppression index (SI; Tadin et al., 2003), which was the log difference of thresholds as a function of stimulus size at low or high contrast (Figure 2C). At high contrast, an ANOVA analysis of the surround suppression indexes revealed that the main effect of age was significant (*F*_(1,14)_ = 7.511, *p* = 0.016, $\eta_{\text{p}}^{2}$ = 0.349), and the main effect of stimulus size was not significant (*F*_(1,14)_ = 0.040, *p* = 0.844, = 0.003). The interaction between stimulus size and age was not significant (*F*_(1,14)_ = 3.496, *p* = 0.083, $\eta_{\text{p}}^{2}$ = 0.200). Simple effect analysis showed that the suppression indexes of 4 degree stimulus were significantly different between the old subjects and the young subjects (*F*_(1,14)_ = 9.884, *p* = 0.007, $\eta_{\text{p}}^{2}$ = 0.414). However, the suppression indexes of 2 degree stimulus were not significantly different between the old subjects and the young subjects (*F*_(1,14)_ = 2.673, *p* = 0.124, $\eta_{\text{p}}^{2}$ = 0.160). *Post hoc* tests (Bonferroni-Holm correction) were conducted and found that SI of 4 degree condition was significantly smaller in the old group than in the young group (*t*_(14)_ = −3.144, *p* = 0.015). As indicated in the study of Sysoeva et al. (2017), the surround SI calculated as the log difference between the thresholds for large stimulus and the thresholds for small stimulus might be affected by both thresholds. We conducted the ANCOVA analysis on the thresholds of 4 degree stimulus while using the thresholds of 1 degree stimulus as the covariate between the elderly and the young subjects. The results revealed that the difference between the elderly and the young subjects was significant (*F*_(1,13)_ = 7.130, *p* = 0.019, $\eta_{\text{p}}^{2}$ = 0.354), suggesting that the surround suppression was reduced for the elderly compared to the young adults. Therefore, all these results suggest the reduced surround suppression in elderly.

## Discussion

Visual cortical processing is based on the interaction of excitation and inhibition across neurons (Alitto and Dan, 2010; Isaacson and Scanziani, 2011). Previous studies have suggested that aging-related visual deficits are the result of reduced inhibition in the visual cortex (Betts et al., 2004; Fu et al., 2010; Bocheva et al., 2013). However, there are different inhibitory mechanisms in the visual cortex. We found that motion/direction repulsion effect was increased with aging. Because motion/direction repulsion has been interpreted as arising from local mutual inhibition (Marshak and Sekuler, 1979; Mather and Moulden, 1980; Qian and Geesaman, 1995; Perry et al., 2014), which is that two groups of neurons with different preferred directions inhibit each other, our result suggests that aging may lead to enhanced local mutual inhibition. Although this result may be initially surprising, it is understandable considering the complexity of the brain’s inhibitory system and neural mechanisms that are affected by aging. For example, Pinto et al. (2010) investigated how GABAergic mechanisms develop in the human visual cortex across one’s lifespan and found that the expression of eight pre- and post-synaptic GABAergic markers change differently during aging.

Although there is still debate surrounding the underlying neuronal mechanisms of motion repulsion, the mainstream view is that it reflects local mutual inhibition between the two population of neurons representing the different directions (Marshak and Sekuler, 1979; Mather and Moulden, 1980; Hiris and Blake, 1996). We generally associate a group of neurons with a particular stimulus condition to emphasize their functional role in coding information. For example, neurons encoding 45-degree motion direction are those that fire most when a stimulus moving at 45 degrees is presented within their receptive field. All neurons have a dynamic response range. The dots moving at 45 degrees excite not only neurons that encode this particular motion direction but also those encoded by “nearby” stimulus conditions. This will decrease our perceptual sensitivity when the neural system utilizes all neuronal activities available that respond to a stimulus. One way to increase perceptual sensitivity is to weaken inaccurate neuronal representations through local intra-areal inhibitory connections, which may be the purpose of local mutual inhibition. In our case, when two groups of neurons were simultaneously activated by two separate components of the visual stimulus, an inhibitory interaction between “nearby” group of neurons in the form of motion repulsion was activated. Motion repulsion can be affected by many factors. When the perceived angle of two moving directions was enlarged beyond a certain extent, such as 135 or 180 degrees, motion repulsion began to reduce. Differences in the speeds of two moving dot patterns also affect direction repulsion (Benton and Curran, 2003; Perry et al., 2014). These findings are consistent with the model that motion repulsion results from inhibitory interactions between nearby populations of neurons (Hiris and Blake, 1996; Perry et al., 2014). If true, stronger intra-areal inhibitory interactions could be the result of increased overlap of tuning curves corresponding to nearby populations of neurons. Aging indeed reduces direction selectivity and increases the spontaneous activity of V1 neurons, which in turn, increases the width of the direction tuning curve (Fu et al., 2013). Therefore, the enhanced local mutual inhibition we observed in the elderly may be a result of decreased motion direction selectivity and increased levels of spontaneous activity in the visual cortex.

Previous studies have suggested that there might be several factors contributing to motion repulsion effect. It will be worth discussing how those factors affect the change of motion repulsion with aging. First, Rauber and Treue (1998) reported that reference repulsion can account for portion of motion repulsion effect. Besides the debate on the reliability of reference repulsion (Wiese and Wenderoth, 2008), the neuronal mechanism of reference repulsion phenomena is unclear. The neuronal mechanism of reference repulsion might be through inhibitory effect as well (Simmering et al., 2006), no matter the reference repulsion came from memory recall (Blake et al., 1997) or enhanced neuronal sensitivity at horizontal direction. Second, adaptation might contribute to motion repulsion (Rauber and Treue, 1999). In our experiment, horizontal/reference direction was presented in each trial. This would cause adaptation effects at horizontal direction. Yang et al. (2008) reported that old monkeys exhibited stronger adaptation to visual motion than young monkeys. However, the adaptation weakens the neuronal responses to horizontal motion direction, which makes the inhibitory/repulsion effect from the neurons preferred horizontal motion direction to the neurons preferred other motion direction weaker. Therefore, if adaptation played a dominant role in our experiment, we would observe a stronger motion repulsion effect in young adults, not the other way around. Third, Chen et al. (2005) reported that attention could reduce repulsion. Our experimental task required participants to judge the motion direction of non-horizontal moving dots instead of horizontal moving dots. Verhaeghen and Cerella (2002) reported that no age-related deficits specific to selective attention has been found.

Another possible explanation of the trend in motion repulsion might be the oblique effect, which shows that motion sensitivity is superior at the cardinal directions relative to the oblique directions (Appelle, 1972). However, this is an unlikely explanation for two reasons. First, if the oblique effect is the major cause of the motion repulsion, similar performance should be observed at 45 and 135 degrees. Yet, our results showed significant difference of motion repulsion effect between 45 degree and 135 degree for both the old group (*t*_(20)_ = 11.35, *p* < 0.001) and young group (*t*_(20)_ = 11.31, *p* < 0.001). Second, motion repulsion and the oblique effect involve different aspects of motion perception. Motion repulsion measures the accuracy of subjects’ performance, which represents the difference between the perceived angle and the true angle. In contrast, the oblique effect measures the precision of behavior, which is the repeatability of successive perceiving the actual angle.

The behavioral paradigm of the drifting Gabor we used in this study was first employed by Tadin et al. (2003). Our data show that the discrimination threshold of a high-contrast moving Gabor did not increase with the enlargement of the Gabor patch in old adults. The surround suppression indexes were much smaller in the old group than in the young group. This result indicates that surround suppression decreases in the elderly, which is consistent with the results of Betts et al. (2004, 2005, 2009). It has been proposed that surround suppression is a perceptual correlate of center-surround antagonism within a population of neurons in the middle temporal visual area (Tadin et al., 2003) and that surround suppression can be reduced using transcranial magnetic stimulation (TMS) to temporarily attenuate the MT/V5 area (Tadin et al., 2011). Inactivating the V2 and V3 cortical areas in alert macaques effectively reduced the strength of surround suppression in the V1 for a high-contrast moving grating (Nassi et al., 2013). Previous studies on multiple components of surround modulation have suggested that the surround suppression we measured is major generated by extra-striate feedback connections (Nassi et al., 2014). Therefore, the reduced surround suppression in the elderly may be a result of attenuated cortico-cortical excitatory feedback connections. On the other hand, as the low-contrast stimulus was increased, both groups showed spatial summation, and there was no degradation of spatial summation in the elderly subjects. This result is consistent with the finding that feedback inactivation reduces surround suppression but not summation (Nassi et al., 2014). These changes also suggest that the decrease of surround suppression is not a result of general impairments.

In conclusion, we used motion repulsion and motion discrimination tasks to investigate aging effects on different inhibitory mechanisms across brain areas. We found the enhanced motion repulsion in the old subjects compared to the young subjects, indicating the enhanced local intra-areal mutual inhibition in the old subjects. On the other hand, elderly showed less change in the discrimination threshold when the size of a high-contrast drifting Gabor was increased, indicating the reduced surround suppression compared to the young adults.

## Author Contributions

HD, WC, SK and TZ designed the experiments, wrote and revised the manuscript. HD and WC conducted the experiments and collected the data. HD, WC and SK analyzed the data. HD, WC and TZ prepared Figure 1. HD and WC prepared Figure 2. All authors have reviewed the manuscript.

## Conflict of Interest Statement

The authors declare that the research was conducted in the absence of any commercial or financial relationships that could be construed as a potential conflict of interest.
